# Supplementary material for: Hydrolysis of Oligodeoxyribonucleotides on the Microarray Surface and in Solution by Catalytic Anti-DNA Antibodies in Systemic Lupus Erythematosus
Source: Curr Issues Mol Biol. 2023 Dec 8;45(12):9887–903. doi: 10.3390/cimb45120617 (PMC10742339; doi:10.3390/cimb45120617)
Supplement: Supplementary file 1 [file cimb-45-00617-s001.zip › cimb-2725820-supplementary.pdf]

Supplementary materials

Article

Hydrolysis of oligodeoxyribonucleotides on the microarray surface and in solution by catalytic anti-DNA autoantibodies in systemic lupus erythematosus

Tatiana S. Novikova [1,2], Evgeny A. Ermakov [1,2,*,†], Elena V. Kostina [1,†,] Alexander N. Sinyakov [1], Alexey E. Sizikov [1,3], Georgy A. Nevinsky [1,2], and Valentina N. Buneva [1,2,*]

[1] Institute of Chemical Biology and Fundamental Medicine, Siberian Branch of the Russian Academy of Sciences, 630090 Novosibirsk, Russia;
[2] Novosibirsk State University, Department of Natural Sciences, Novosibirsk 630090, Russia;
[3] Institute of Clinical Immunology, Siberian Branch of the Russian Academy of Sciences, 630099 Novosibirsk, Russia;
* Correspondence: evgeny_ermakov@mail.ru (E.A.E.); buneva@niboch.nsc.ru (V.N.B.)
† These authors contributed equally to this work.

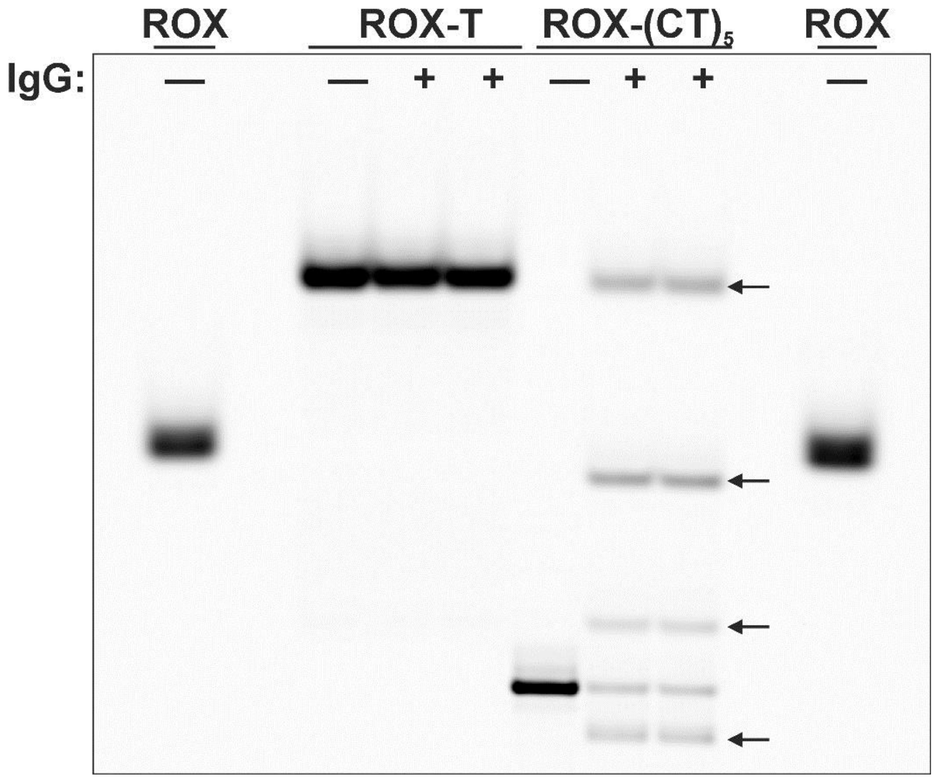

Figure S1. Analysis of the effect of ROX dye on the electrophoretic mobility of ODNs and identification of the final product of hydrolysis by antibodies of SLE patients. ROX dye without nucleotide (lines ROX) migrated to a different location than mononucleotide T labeled ROX (ROX-T). Hydrolysis of decanucleotide (ROX-(CT)$_5$) by IgG preparation SLE33 resulted in the formation of four products (marked by arrows), one of which was presumably a mononucleotide (according to electrophoretic mobility). ROX-T was not hydrolyzed by the IgG preparation SLE33.
